# Dose-dependent effect of canine lyophilized platelet on an in vitro hemodilution model

**DOI:** 10.1186/s12917-023-03614-3

**Published:** 2023-03-17

**Authors:** Mu-Young Kim, Hyun-Jung Han

**Affiliations:** 1 Department of Veterinary Surgery, College of Veterinary Medicine, Konkuk University, 05029 Seoul, Republic of Korea; 2 Department of Veterinary Emergency and Critical Care, College of Veterinary Medicine, Konkuk University, 05029 Seoul, Republic of Korea; 3 KU Center for Animal Blood Medical Science, Konkuk University, 05029 Seoul, Republic of Korea

**Keywords:** Coagulability, Dosage, Platelet function analyzer, Thromboelastography, Transfusion

## Abstract

The transfusion of stored platelets has emerged as an efficient method for treating dogs with thrombocytopenia. However, the availability of fresh platelets is limited in veterinary medicine due to demanding storage conditions. Lyophilized platelets have long shelf lives and can be easily stored, increasing their accessibility for thrombocytopenic dogs. Due to the lack of research and information on the dose effect, canine lyophilized platelets are used at a clinical dosage without research-based evidence. This study was to evaluate the dose effect of lyophilized canine platelets on blood coagulability. Three different concentrations of lyophilized canine platelets were added to in vitro hemodilution blood model, increasing the platelet count by 25, 50, and 100 × 10^6^/ml and coagulation profiles were analyzed. The coagulability was evaluated via the plasma fibrinogen concentration, coagulation time, thromboelastography (TEG), and platelet function analyzer (PFA). Higher concentrations of lyophilized platelets showed dose-dependent association with decreased aPTT and R-time of TEG and increased alpha angle and MA of TEG. These results showed the potential that the higher dose of canine lyophilized platelets better improve blood coagulability than the standard dose and provided the basis for further safety and clinical studies.

## Introduction

Platelet transfusion is used prophylactically or therapeutically for bleeding in thrombocytopenic patients. In particular, it is commonly indicated for thrombocytopenic patients who require invasive procedures. The contributing factors of thrombocytopenia in dogs can be divided into those that cause decreased marrow production, increased destruction, and excessive consumption [1, 2]. Primary bone marrow suppression and low thrombopoietin levels result in a repressed platelet production rate [3]. The causes of increased platelet destruction include primary or drug-induced immune thrombocytopenia (ITP) and infectious diseases such as ehrlichiosis [4]. The increased platelet consumption occurs in disseminated intravascular coagulation and massive blood loss [5]. In clinical practice, ITP is the most common cause of canine thrombocytopenia [6]. The usage of platelet transfusion in ITP patients is controversial, as transfused platelets were demonstrated to be rapidly destroyed after administration [1, 6, 7]. However, for dogs with uncontrolled and life-threatening hemorrhage, platelet transfusion is recommended, although the duration of the hemostatic effect is limited [1].

In veterinary medical practice, the lack of readily available canine platelet products is the major limitation for platelet transfusion. Due to the difficulties in collecting and storing canine platelets, the transfusion of platelets is mainly limited to platelets contained in fresh canine whole blood [8]. Various modalities have been devised to create canine platelet products to increase the clinical applicability of canine platelets and overcome practical limitations, including a lack of blood donors, low production efficiency, and short shelf life [6, 9–11]. Recently, developed lyophilized canine platelet products showed several advantages in terms of shelf life and preservation of platelet function [9, 12]. By using the natural cryoprotectant trehalose, canine platelets can be lyophilized without severe structural and functional deformation [13]. Trehalose agents stabilize platelet membranes and prevent the formation of damaging ice crystals by replacing water within the platelets [14]. In contrast to fresh platelets, which have a shelf life of 5 days, lyophilized platelets can be stored for up to 2 years [1, 15].

In general, one unit of fresh platelets (6 ~ 8 × 10^10^ platelets), platelet-rich plasma (PRP) or platelet concentrate (PC), per 10 kg body weight has been recommended as a proper dose for thrombocytopenic dogs, which theoretically raises the recipient’s platelet count by 40 × 10^6^/ml [16]. In the case of cryopreserved or lyophilized platelets, a substantially greater total number of transfused platelets is required to achieve a similar platelet increment compared to fresh platelets because many platelets are lost by freeze–thaw and lyophilization processes [13, 17–19]. In addition, after being transfused, cryopreserved or lyophilized platelets are cleared from the free circulation faster than fresh platelets [20, 21]. Therefore, the authors hypothesize that the optimal transfusion dose of lyophilized platelet is higher than that of fresh platelets.

Only a limited number of studies have been conducted on canine lyophilized platelets [12, 22]. In particular, there are no studies assessing the dose effects of canine lyophilized platelets. The aim of the present study was to evaluate the effect of differences in transfusion doses of lyophilized canine platelets on blood coagulability and, based on the results, to establish a basis for the clinical application of canine lyophilized platelets. Several doses of canine lyophilized platelets were added to an in vitro hypocoagulation blood model made by diluting canine whole blood with normal saline, and coagulation profiles were analyzed.

## Materials and methods

### Animals

Eight male beagle dogs (20 months old), weighing 12–14 kg, were purchased from a commercial laboratory for experimental use. Dogs were clinically healthy and did not take any medications that could affect the coagulability of the blood, such as antithrombotic, anti-inflammatory, and antiplatelets. The results of the blood tests, including complete blood counts, serum electrolytes and chemistries, and coagulation profiles were within the reference ranges in all dogs. This study was approved by the Institutional Animal Care and Use Committee at Konkuk University (Approval number KU21059). After completion of the study, all eight dogs were adopted to private homes.

### Preparation of lyophilized platelets

**Fig. 1:**
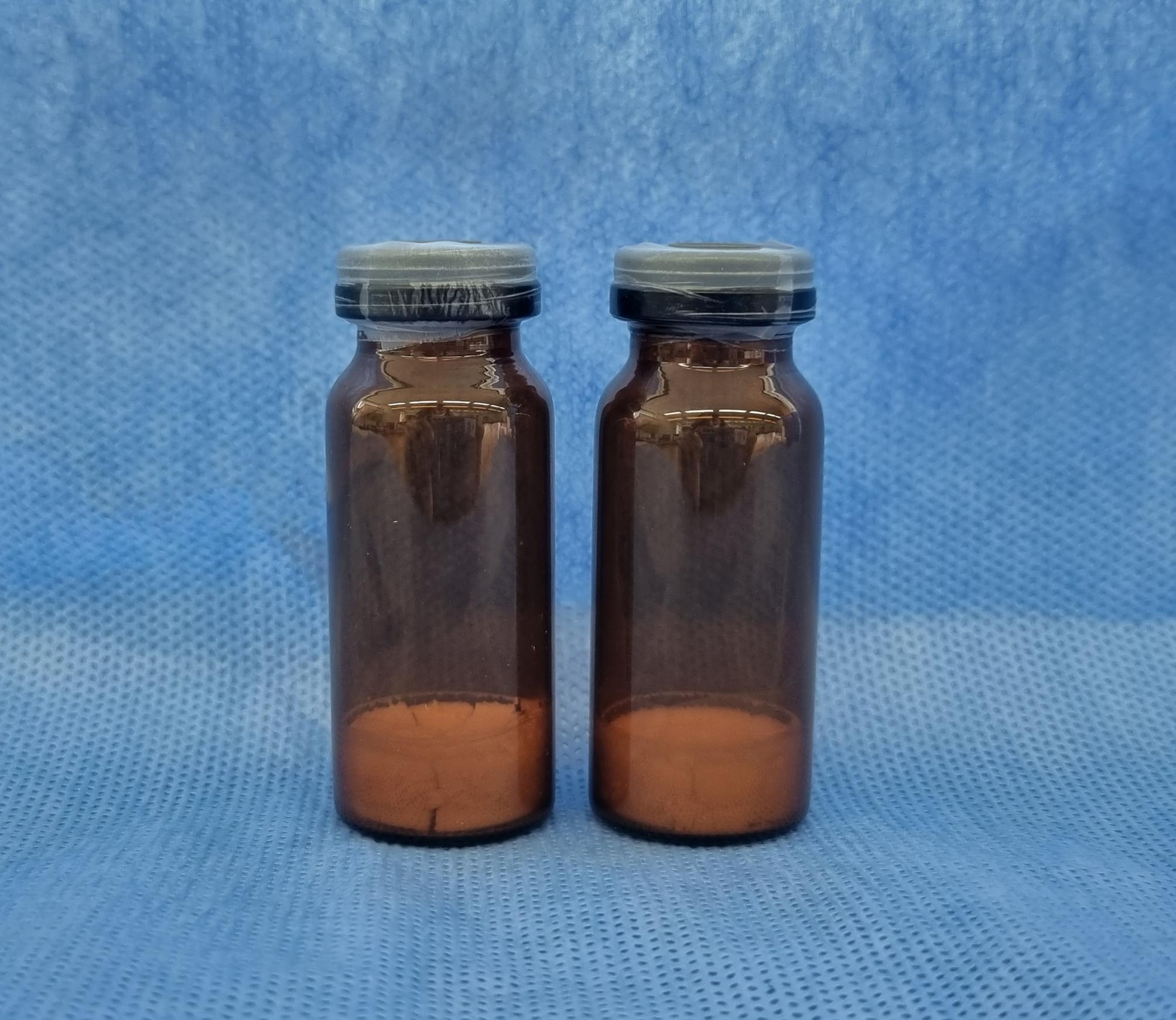
Vials of lyophilized platelet powder. Sterile distilled water is added to reconstitute the platelet powder before use in the experiments

Whole blood (90 mL each) was obtained from the eight beagle dogs using a 20-gauge intravenous catheter and transferred into 50 mL syringes containing 5 mL of 3.2% w/v sodium citrate. The final dilution ratio of blood:sodium citrate was 9:1 (v/v). The blood samples were centrifuged at 900×g for 5 min to harvest platelet concentrate (PC). The PCs of the eight dogs were pooled together and analyzed for complete blood count. The pooled PC was centrifuged at 1250×g for 10 min. The platelet pellet was resuspended in Tyrode’s Buffer (9.5 mM HEPES, 100 mM NaCl, 4.8 mM KCl, 12 mM NaHCO_3_, 10 µg/µl prostaglandin E1, and 1% ethanol) containing 50 mM trehalose, pH 6.8, and the platelet count was adjusted to 1 × 10^9^/ml, based on the complete blood count results of the PC. The platelet solution was incubated at 37 °C for 4 h and stirred every 30 min during incubation. Bovine serum albumin was added to a final concentration of 5%. The platelet solution was transferred into glass vials with rubber caps and subsequently frozen from 22 to -40 °C with a freezing rate of -2 °C/minute. After freezing, the frozen platelet solutions were transferred onto the shelf of a programmable freeze-drier (LP03, ilShinBioBase, Dongducheon, Korea) with a shelf temperature of -40 °C. Primary drying was performed at -40 °C under 20 mTorr for 16 h, and during secondary drying, the shelf temperature was increased up to 22 °C at a rate of 0.2 °C/minute. The samples were kept at 22 °C for 16 h. Throughout the whole lyophilization process, the vacuum was maintained at 4 mTorr. After the lyophilization process was complete, the samples were sealed with rubber caps and stored at room temperature (Fig. 1).

The aggregation capacity of canine lyophilized platelets was evaluated using light transmission aggregometry (LTA, Chronolog 490, Chrono-Log Corp., PA, USA) and monitored by using Agrolink software (Chrono-Log Corp., PA, USA). Lyophilized platelet samples were reconstituted with sterile water to prepare lyophilized platelet solution (LPS) prior to analysis. LTA was performed on fresh PC and LPS, which were pooled from the eight dogs. To perform the LTA test, 500 µl (200 × 10^6^ plts/ml) of PC and LPS were used. Each sample was placed in a cuvette with a stirring bar magnet and preincubated at 37 °C in the presence of 1 mM calcium chloride. An ADP agonist (Chrono-Log Corp., PA, USA) was added to a concentration of 10 µM, and the aggregation curve was recorded for 10 min. The LTA optical detection system is based on changes in turbidity. The increase in light transmission is proportional to the degree of platelet aggregation. Therefore, platelet aggregation was quantified by measuring the light transmittance (%). The test was repeated five times on PC and LPS samples, and each mean value was calculated. The maintenance of the platelet aggregation response after lyophilization was analyzed by comparing these mean values.

### Study design and classification of the experimental groups

Whole blood was drawn from the jugular vein of each beagle dog directly into the vacutainer tube containing 3.2% sodium citrate using a 20-gauge butterfly needle. The ratio of whole blood to anticoagulant was 9:1 (v/v). From a single blood collection, the whole blood samples were divided into four groups: one control group and three experimental groups. In the control group, the whole blood samples were diluted 1:1 with normal saline. The lyophilized platelet samples were reconstituted with distilled water to their original volumes. Based on the platelet count before lyophilization, LPS was diluted with normal saline to concentrations of 50, 100, and 200 × 10^6^ plts/ml. In the experimental groups, three different concentrations of LPS were mixed 1:1 with the whole blood samples to increase the platelet count of the hypocoagulation blood model by 25 (Group 1), 50 (Group 2), and 100 (Group 3) ×10^6^/ml compared to the control group. This study design represents the transfusion of lyophilized platelets into a dog with severe hemorrhage or hypocoagulability.

The experimental design described above was repeated in eight beagle dogs. Coagulation profiles were analyzed to assess the dose effects of lyophilized platelets via plasma fibrinogen concentration, prothrombin time (PT), activated partial thrombin time (aPTT), thromboelastography (TEG), and platelet function analyzer (PFA). All tests, except TEG, were performed immediately after the diluted blood samples were prepared. TEG tests were performed after 30 min after the blood collection.

### Plasma fibrinogen concentration and coagulation times

The plasma concentration of fibrinogen and coagulation times (PT, aPTT) were measured using a coagulation analyzer (CG02NV, A&T Corp., Tokyo, Japan). The concentration measurement of fibrinogen, the major plasma protein coagulation factor, is for evaluating the fibrinogen residues in the lyophilized platelet samples. The PT is a screening test for evaluating coagulation factors involved in extrinsic and common pathways. The aPTT is a screening test for coagulation factors of intrinsic and common pathways. Prolonged PT and aPTT indicate deficiencies or inhibition of extrinsic and intrinsic coagulation factors. Plasma samples were obtained from blood samples by centrifugation at 3000×g for 10 min. Each plasma sample was used for fibrinogen, PT, and aPTT analyses.

### Thromboelastography

TEG was performed for the viscoelastic measurement of clot formation, providing information on the overall blood coagulation status. The clotting potential of blood samples after kaolin activation was evaluated by TEG (TEG® 5000, Hemonetics Corp., MA, USA). The TEG process was performed with 340 µL of kaolin-activated blood samples and 20 µL calcium chloride, following the manufacturer’s recommendations. The variables recorded were R time (time until initial formation of clot), K time (time until clot reaches 20 mm from the start of clot formation, rapidity of clot formation), α angle (angle between the baseline and a tangent line intersecting the tracing line, speed of clot formation), and MA (maximal amplitude, strength of the clot). The reference ranges for these parameters were determined based on the study by Bauer et al. [23].

### Platelet function analyzer

The hemostatic activity of each blood sample was evaluated by using PFA (PFA-200, Seimens Health care, Marburg, Germany). The cartridge collagen/adenosine diphosphate (CADP) was used to measure the closure time (Ct) for platelet aggregation to occlude a miro-aperture in a CADP-coated membrane under vacuum. A total of 900 µL of blood sample was used for the PFA assay. Based on the studies by Burgess and Saati et al., the reference range for CADP was set as 45–111 s [24, 25].

### Statistical analysis

The test values of plasma fibrinogen concentration, PT, aPTT, TEG variables, and PFA were analyzed to evaluate the dose effects of canine lyophilized platelets using the nonparametric Kruskal–Wallis test, followed by the Mann–Whitney post hoc test for multiple comparisons. All the experimental values are presented as the median and range. A *P* value under 0.05 was considered statistically significant. Statistical analysis was performed using IBM SPSS (v25.0, SPSS Inc., IL, USA).

## Results

**Fig. 2:**
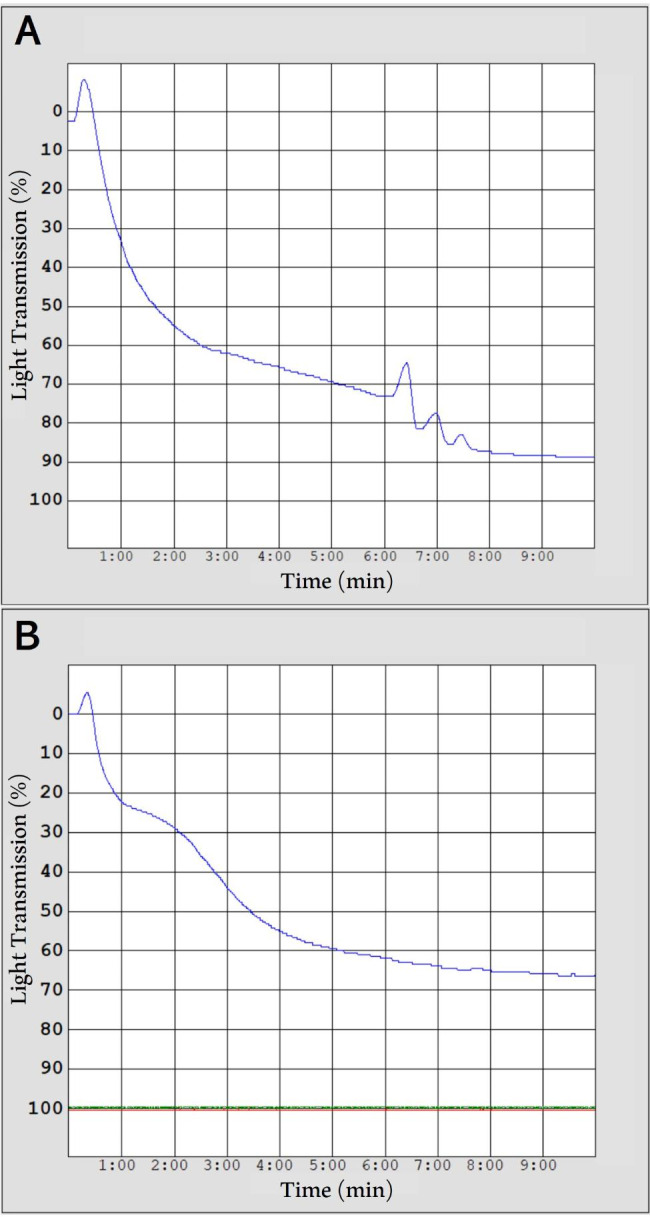
Representative light transmission aggregation traces of fresh platelet-rich plasma (A) and lyophilized platelet solution (B). The magnitude of platelet aggregation is recorded as % light transmittance. The light transmission percentage at 10 min was measured in response to adenosine diphosphate (10 µM)

The hematocrit and platelet count for all dogs enrolled in this study were within the reference interval. The median hematocrit and platelet count of the control group blood samples were 19.75% (16.8 − 22.3%) and 155 × 10^6^ (132 × 10^6^ − 183 × 10^6^) plts/mL, respectively. The mean LTA values of PC and LPS were 88.4% and 71.2%, respectively (Fig. 2). Table 1 shows the coagulation test results for the control group and three experimental groups in which lyophilized platelets were supplemented.

**Table 1:** Data summary of coagulation profiles

		Control	Group 1	Group 2	Group 3
Fibrinogen (mg/dl)		76 (55–89)	78 (63–94)	77 (62–97)	81 (60–96)
PT (sec)		9.05 (8.6–9.3)	9.1 (8.7–9.5)	9.15 (8.8–9.5)	9.05 (8.9–9.4)
aPTT (sec)		32.5 (28.9–37.0)	27 (25.1–32.4)	24.6 (21.9–27.3)^*^	24.7 (19.3–30.3)^*^
TEG	**R time (min)**	4.1 (2.8–5.2)	2.15 (1.5–2.8)^*^	1.8 (1.1–2.5)^*^	1.75 (1.3–2.1)^*^
	**K time (min)**	0	0 (0-7.8)	0 (0-4.5)	3.5 (0–7)
	**Angle (°)**	34.3 (21.1–58.3)	49.7 (37.1–67.1)^*^	54.5 (44.3–70)^*^	54.7 (42.6–64.6)^*^
	**MA (mm)**	8.7 (3.1–16.1)	11.2 (6.1–23.4)	15.4 (10-24.6)^*^	25.2 (172 − 31.5)^*#^
Ct (sec)		210 (139–287)	169 (131–219)	160 (139–195)	145 (118–162)^*^

Data are expressed as the median (range). ^*^Asterisk indicates a significant difference compared to the Control group (p < 0.05). ^#^Hash indicates a significant difference compared to the Group 2 (p < 0.05). Statistical analysis was not performed for K times due to the insufficient amount of data. No lyophilized platelets were added to the control group. The canine lyophilized platelets were added to the amount that could increase the platelet count by 25 (Group 1), 50 (Group 2), and 100 (Group 3) ×10^6^/ml

PT = prothrombin time; aPTT = activated partial thrombin time; MA = maximum amplitude; Ct = closure time; plts = platelets

### Fibrinogen

**Fig. 3:**
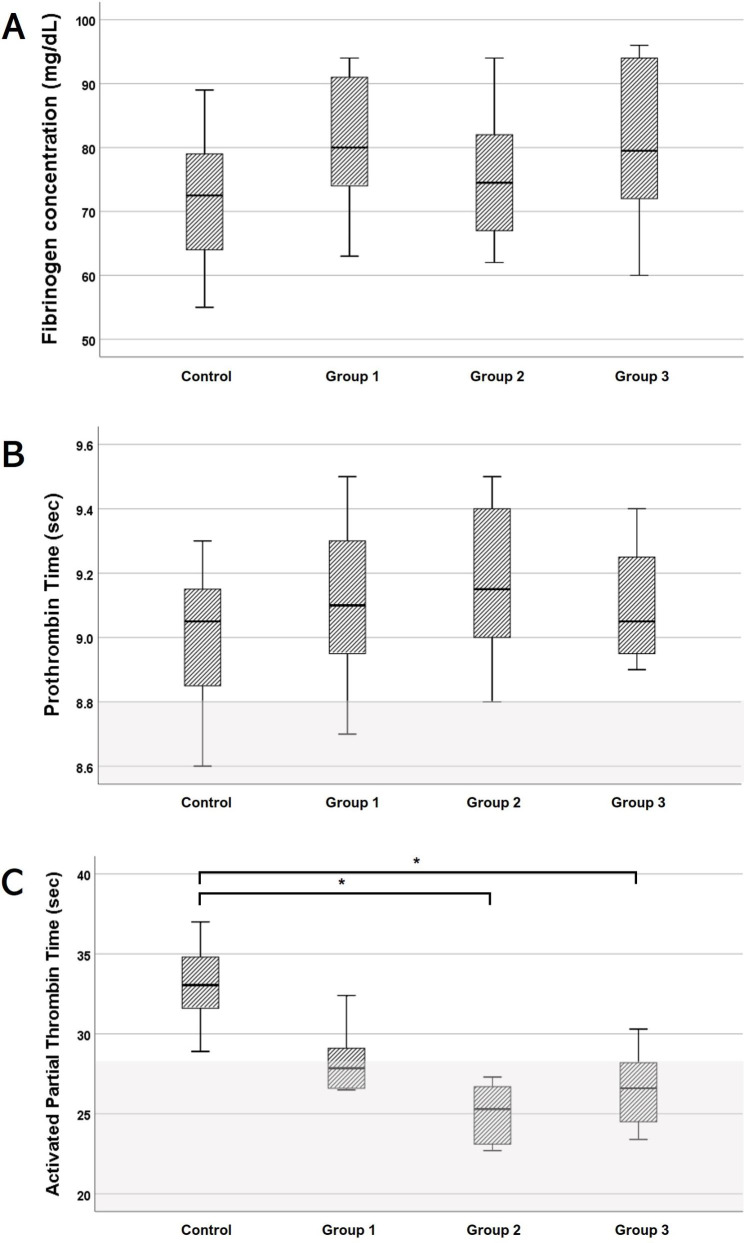
Correlation of lyophilized platelet supplementation dose with fibrinogen concentration (A), prothrombin time (B), and activated partial thrombin time (C). No lyophilized platelets were added to the control group. The canine lyophilized platelets were added to the amount that could increase the platelet count by 25 (Group 1), 50 (Group 2), and 100 (Group 3) ×10^6^/ml. ^*^Asterisk over bracket indicates statistical significance between the two groups (p < 0.05). The shaded area indicates the reference range. The plasma fibrinogen concentration was below the reference range (150–350 mg/dl) in all groups

In all groups, the plasma fibrinogen concentration was below the reference interval (150–350 mg/dl). No significant differences were found between all four groups. Canine lyophilized platelet supplementation did not significantly increase the plasma fibrinogen concentration (Fig. 3A).

### Prothrombin time and activated partial thrombin time tests

All the groups revealed prolonged PT and aPTT (reference range: PT = 7.4–8.8 s, aPTT = 12.0–28.0 s) (Fig. 3B, C). PT results showed no significant difference between the groups. For aPTT, Groups 2 and 3 showed significantly reduced values compared to the control group (p < 0.05). In contrast, there was no significant difference in aPTT between the control group and Group 1.

### Thromboelastography

**Fig. 4:**
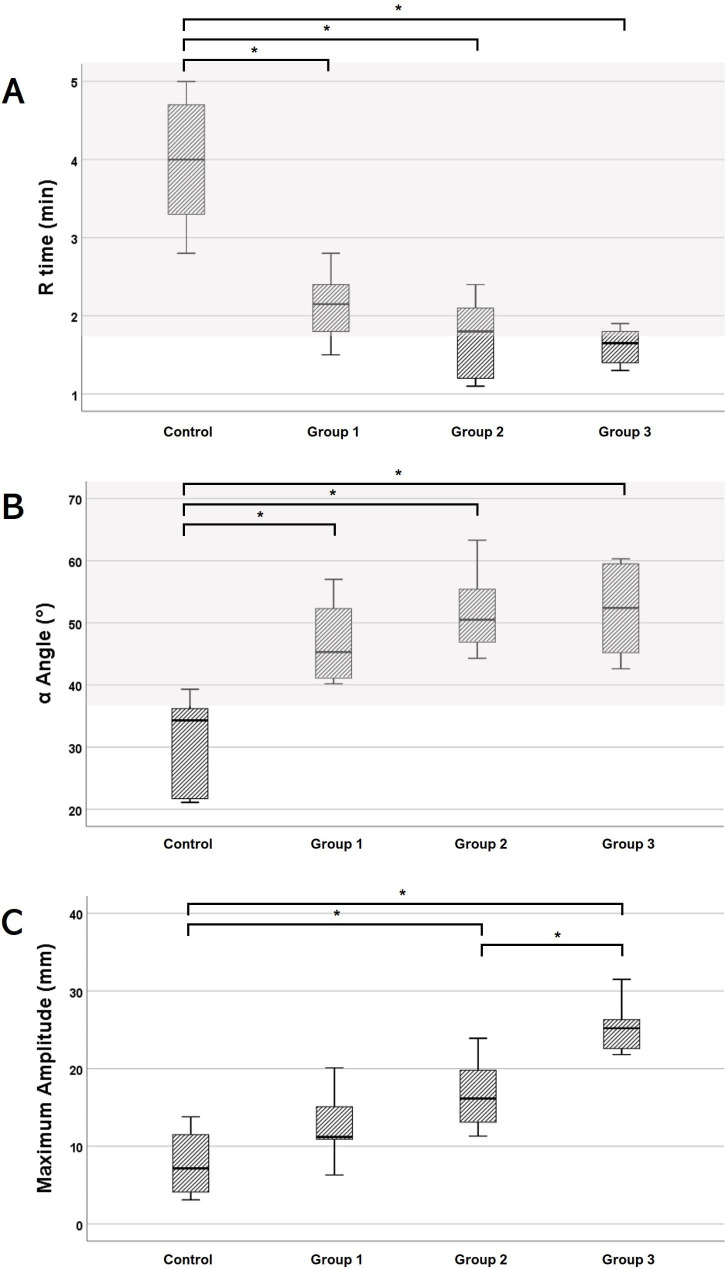
Correlation of lyophilized platelet supplementation dose with thromboelastography variables: R time (A), α angle (B), and maximum amplitude (C). No lyophilized platelets were added to the control group. The canine lyophilized platelets were added to the amount that could increase the platelet count by 25 (Group 1), 50 (Group 2), and 100 (Group 3) ×10^6^/ml. ^*^Asterisk over bracket indicates statistical significance between the two groups (p < 0.05). The shaded area indicates the reference range. The maximum amplitude was below the reference range (42.9–67.9 mm) in all groups

The R time was significantly lower in the three lyophilized platelet-supplemented groups (Groups 1, 2, and 3) than in the control group (p < 0.05) (Fig. 4). No significant difference was found between these three experimental groups. The mean R time value was within the reference range (1.8–8.6 min) in control and Group 1. Groups 2 and 3 revealed lower mean R time values than the reference range. The K time was recorded only when MA reached 20 mm. None of the control group samples reached 20 mm of MA; therefore, no K time was recorded in the control group. Only two and three K time values were obtained from Groups 1 and 2, respectively. All samples of Group 3 reached 20 mm of MA and revealed a K time value. The mean K time value of Group 3 was within the reference range (1.3–5.7 min). The experimental groups showed significantly higher values of α angle than the control group (p < 0.05) and revealed mean α angle values that were within the reference range (36.9–74.6 degrees). There was no significant difference in the α angle between the experimental groups. The mean α angle value of the control group was lower than the reference range. The MA values of Groups 2 and 3 were significantly higher than those of the control group (p < 0.05). The dose increase of lyophilized platelets from Group 1 to Group 3 induced a significant increase in MA (p < 0.05). In all four groups, the mean MA values were less than the reference range (42.9–67.9 mm).

### Platelet function analysis

**Fig. 5:**
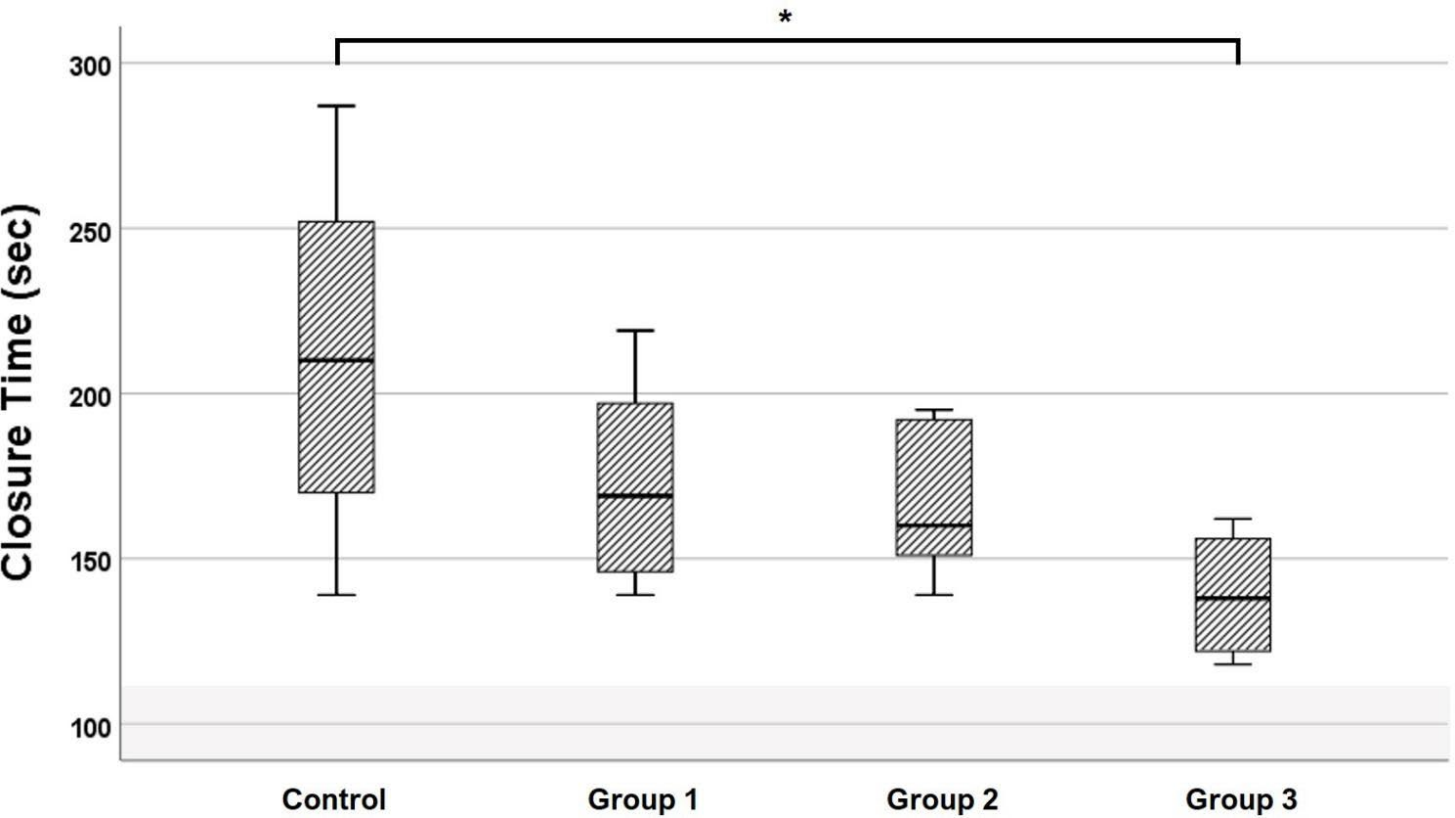
Correlation of lyophilized platelet supplementation dose with platelet function analyzer closure time. No lyophilized platelets were added to the control group. The canine lyophilized platelets were added to the amount that could increase the platelet count by 25 (Group 1), 50 (Group 2), and 100 (Group 3) ×10^6^/ml. ^*^Asterisk over bracket indicates statistical significance between the two groups (p < 0.05). The shaded area indicates the reference range. The closure time was over the reference range (45–111 s) in all groups

The Ct value decreased progressively as the concentration of lyophilized platelet solution increased; however, a significant difference was found only between control and Group 3 (p < 0.05) (Fig. 5). The mean Ct values of all groups were over the upper limit of the reference range.

## Discussion

In human medicine, studies on the appropriate platelet transfusion dose have been actively conducted and guidelines were developed [26, 27]. Based on these data, approximately 3 × 10^11^ to 6 × 10^11^ plts (6 units of whole-blood derived platelets or 1 unit of apheresis platelets) per person, which approximately equals to 2 ~ 4 × 10^11^/m^2^ in 60 kg adult, is considered a standard clinical dose [28–30]. The conversion from kg to m^2^ was performed based on the body surface area fomula [31]. In one study, low-dose transfusion (1.1 × 10^11^ plts/m^2^) had similar efficacy in preventing bleeding as medium-dose (2.2 × 10^11^ plts/m^2^) and high-dose (4.4 × 10^11^ plts/m^2^) transfusions [28]. In addition, low-dose transfusions have been shown to reduce the total number of platelets transfused in a single patient [28]. As such, the optimal dose of platelet transfusion is controversial even in human medicine. The platelet transfusion protocols in veterinary clinics have been adopted from human studies; however, no studies have validated whether these protocols, especially transfusion doses, are suitable for dogs and cats. In this study, to compare the clinical transfusion dosages between human and dog, the platelet count parameters (unit, /person, /kg) were converted as needed into /m^2^.

The lyophilization process impaired the function of canine platelets in the present study. The responsiveness of platelets to ADP was markedly decreased after lyophilization. These results were consistent with previous literature suggesting impairment of platelet signaling and downstream responses by lyophilization [32]. In addition, various studies have demonstrated that lyophilized canine platelets can be removed from free circulation much more rapidly than fresh platelets due to the desialylation mechanism [7, 20, 21, 33]. Considering these limits, it is unreasonable to directly apply a human platelet transfusion protocol to canine lyophilized platelet transfusion. Therefore, to maximize the therapeutic or prophylactic effect of platelet transfusion, a transfusion protocol suitable for canine lyophilized platelets should be formulated.

To indirectly evaluate the dose effect of canine lyophilized platelet transfusion, a hypocoagulation blood model was used in this study. The blood model was made by diluting whole blood 1:1 with normal saline, and as a result of complete blood count and several coagulability evaluation tests, it was confirmed that all values were lower than the normal values. This blood model serves as the control group, simulating a hemorrhaging patient’s blood makeup, where hematocrit, platelet count, and coagulation factors are significantly depleted. The degree of change in coagulability was measured by adding lyophilized platelets at various concentrations to the hypocoagulation blood model. In general, the transfusion dose of canine lyophilized platelets used in veterinary clinical practice is 3 ~ 4 × 10^9^/kg, which is approximately 0.5 ~ 1.5 × 10^11^/m^2^ in dogs with 5 ~ 50 kg of body weight. Considering that the actual recovery rate of transfused fresh autologous platelets is 51% and the average estimated blood volume is 80 mL/kg in dogs, a standard transfusion concentration of 3 ~ 4 × 10^9^/kg would theoretically result in a maximum platelet increment of 20 ~ 25 × 10^6^/ml [34]. This transfusion dose is lower than the standard fresh platelet transfusion dose for dogs, 6 ~ 8 × 10^9^/kg, and humans, 3 ~ 6 × 10^11^/person [16, 28, 29]. Although there is a study result [28] showing that the dose of 1.1 × 10^11^/m^2^ has a similar effect in preventing bleeding compared to the higher dose of 2.2 × 10^11^/m^2^ or 4.4 × 10^11^/m^2^, considering that the function of lyophilized platelets is lower than that of fresh platelets and, in comparison to humans, the expected platelet increment in dogs is lower due to the lower recovery rate of transfused platelets and higher blood volume in dogs. We hypothesized that administration of a higher dose of canine lyophilized platelets would be necessary for optimal therapeutic or prophylactic effects. The coagulability was compared and analyzed by adding canine lyophilized platelets in an amount that could increase the platelet count of the hypocoagulation blood model by 0, 25, 50, 100 × 10^6^/ml.

Fibrinogen concentration values showed no statistically significant difference between all groups, which indicates that no significant amount of fibrinogen was left in the process of platelet lyophilization. There was no significant difference between the groups in the PT test; in contrast, a significant improvement in coagulability was confirmed in Group 3 compared to the control group in the aPTT test. These results suggest the possibility that the lyophilized platelet sample contains significant amounts of intrinsic coagulation factors. However, coagulation factors were not measured in this study. Another possibility is that the aPTT test is more sensitive to the increase in platelet count than the PT test. Further research is needed to determine the exact cause.

The TEG test was performed to evaluate the effect of lyophilized platelet transfusion on the overall coagulation process. No significant difference in R time was found between the groups; however, in Group 2 and Group 3, clot formation started faster than the normal range, in contrast to the control group and Group (1) In the case of K time, the difference between groups was especially clear because there were specific conditions for K time to be recorded (MA > 20 mm). In the other groups, only a few or no samples formed clots of sufficient strength, whereas in Group 3, all samples showed clot strength greater than 20 mm. The median value was 0 in control group, Group 1, and Group (2) Because K time was not recorded in many samples as described above, statistical analysis of K time, which represents the speed of clot formation, was not performed. The speed of clot formation was analyzed through the α angle, which offers similar information to the K time. The control group without lyophilized platelet supplementation showed an α angle lower than the normal range, but all groups with lyophilized platelet supplementation were within the normal range and showed significant differences compared to the control group. However, there was no significant difference in the speed of clot formation according to the dose of supplemented lyophilized platelets. The MA value, which directly evaluates the strength of the clot, showed an increasing trend as the dose of lyophilized platelets increased, even though there was no significant difference between groups. Langhorn et al. reported that whole blood and fresh frozen plasma also lead to improvement in TEG parameters [35]. The results of these TEG test variables showed that even when canine lyophilized platelets are administered at a higher dose than the standard platelet transfusion dose, coagulability could be improved as the administered dose is increased.

The PFA test, which evaluates the coagulation properties of platelets in blood samples in response to platelet agonists, demonstrated that only Group 3, to which the highest dose of platelets was added, had a significant difference compared to the control group. These results showed that transfusion of lyophilized platelets at a standard dose, commonly used in veterinary clinical practice, may not induce a significant level of coagulation improvement in dogs with severe hemorrhage or hypocoagulation. In this study, only one agonist (collagen/adenosine diphosphate) is used in PFA. Additional PFA tests with other agonist (collagen/epinephrine) may reinforce the results.

As a result of the comprehensive analysis of the coagulation profiles above, it is clearly confirmed that coagulability is improved when canine lyophilized platelets are supplemented. However, each evaluation variable showed different results as to whether the supplementation of lyophilized platelets at a higher dose than the standard dose had significant improvement in coagulability. The aPTT, R time, K time, MA, and Ct results showed that the administration of canine lyophilized platelets at a higher dose than the standard dose was significant; however, there was no significant difference in fibrinogen concentration, PT, or α angle. In the author’s opinion, transfusion of canine lyophilized platelets at a higher dose than the standard dose currently used in veterinary clinics may show better therapeutic or prophylactic effects than the existing lyophilized platelet transfusion protocol. However, further in vivo study should be performed to clarify this point.

Because this study has the limitation of using a hypocoagulation blood model, it is necessary to evaluate the dose effect in dogs with coagulation disorders through in vivo studies. In addition, studies on the side effects of lyophilized platelets in dogs should be conducted. In human medicine, it has been reported that platelet transfusion has various potential adverse reactions and, among them, the leukocyte proinflammatory mediator, which progressively increases during the storage of platelets, and bacterial contamination is considered the main cause [36, 37]. Proinflammatory cytokines are produced by platelets and leukocytes in platelet products, and bacterial contamination is mainly caused by skin bacteria during phlebotomy [37, 38]. The risk of these two factors increases during platelet storage [39]. These side effects can be minimized in lyophilized platelets because unlike fresh platelets, lyophilized platelets undergo several washing processes to remove inflammatory substances in plasma, and infectious agents can be eliminated through radiation sterilization after lyophilization. However, there is a possibility that the rejection reaction is induced by additives used for platelet lyophilization and that various infectious agents are introduced during the lyophilization preparation process. Therefore, the optimal dose of canine lyophilized platelets in dogs should be established to maximize the therapeutic effect while minimizing side effects based on the study results on canine lyophilized platelets. To date, no studies have analyzed the side effects of lyophilized platelets in human or veterinary medicine.

In conclusion, this study suggests potential benefits of transfusion dose escalation of canine lyophilized platelets. However, further in vivo safety and efficacy studies with carefully controlled clinical settings should be conducted to confirm whether a higher transfusion dose of canine lyophilized platelets in dogs is of practical significance. The authors expect this study to inspire more studies to explore the dose effect of a canine lyophilized platelet.

## Data Availability

The datasets that support the findings of this study are available from the authors upon reasonable request.

## Abbreviations

ITP: immune thrombocytopenia
PRP: platelet-rich plasma
PC: platelet concentrate
PT: prothrombin time
aPTT: activated partial thrombin time
TEG: thromboelastography
PFA: platelet function analyzer
CADP: catridge collagen/adenosine diphosphate
Ct: closure time
SD: standard deviation
